# Acute severe hypoglycemia immediately after induction of anesthesia in an elderly patient with type 2 diabetes mellitus: A case report

**DOI:** 10.1097/MD.0000000000036683

**Published:** 2023-12-22

**Authors:** Qin Tian, Ming Liang Yi, Jia Lu Wan, Hong Yin

**Affiliations:** a Department of Anesthesiology, Chengdu Fifth People’s Hospital (The Second Clinical Medical College, Affiliated Fifth People’s Hospital of Chengdu University of Traditional Chinese Medicine), Chengdu, China.

**Keywords:** diabetes mellitus, general anesthesia, hypoglycemia, opioids

## Abstract

**Rationale:**

Acute severe hypoglycemia immediately following anesthesia induction is a rare but life-threatening complication that is frequently underdiagnosed due to insufficient awareness. Among the various physiological processes influenced by opioids, alterations in blood glucose levels induced by opioids are a side effect that is commonly overlooked. The significance of this report lies in emphasizing the neglected association between opioids and hypoglycemia and highlighting the importance of close glucose monitoring to prevent hypoglycemic events in the perioperative setting.

**Patient concerns:**

An 89-year-old man with type 2 diabetes mellitus experienced acute severe hypoglycemic episode immediately after general anesthesia induction. Baseline blood glucose level before starting anesthesia induction was 4.0 mmol/L. However, it decreased substantially to 0.96 mmol/L immediately after anesthesia induction.

**Diagnosis:**

The patient exhibited normal serum insulin, C-peptide, and cortisol levels, alongside unremarkable renal and hepatic function. After excluding other causes of hypoglycemia, we speculate that opioids were the culprits due to the temporal association and the rapid decline in blood glucose levels.

**Interventions:**

Forty milliliters of 50% dextrose were administered intravenously followed by an infusion of 5% dextrose.

**Outcomes:**

Recovery from anesthesia, extubation, and postoperative recovery were unremarkable. No further hypoglycemic episodes occurred during hospitalization.

**Lessons:**

A precipitous and rapid decline in blood glucose following anesthesia induction is extremely uncommon. When a clinical anesthesiologist detects an abnormally low bispectral index during general anesthesia, hypoglycemia should be suspected. Instituting glucose monitoring in these situations can enable a timely diagnosis, forestalling the onset of life-threatening severe hypoglycemia.

## 1. Introduction

Diabetes mellitus (DM) is a widespread chronic disease that is becoming increasingly prevalent globally, with an estimated 537 million people currently affected.^[1]^ More and more individuals with diabetes will receive anesthesia and surgery. Acute severe hypoglycemia during general anesthesia induction is a rare but serious complication in patients with type 2 diabetes mellitus (T2DM). Hypoglycemia can lead to neurological deficits, cognitive decline, and even neurological damage.^[2]^ It may also result in arrhythmias, myocardial ischemia, and other cardiovascular events.^[3]^ Hypoglycemia induced by analgesics such as tramadol,^[4–7]^ methadone,^[8,9]^ and morphine^[10]^ has been reported in the literature. However, hypoglycemia in patients during general anesthesia induction with opioids has not been previously reported. The purpose of this case report is to describe an unusual presentation of abrupt fall in blood glucose after opioid general anesthesia induction in an elderly patient with T2DM. Thus, it is very important to increase awareness of the potential for opioid-associated hypoglycemia in elderly T2DM patients undergoing general anesthesia and promote improved anesthesia management strategies to mitigate hypoglycemia-related complications. This report also provides ground for future studies about hypoglycemia as a possible adverse event associated with opioid use.

## 2. Case presentation

An 89-year-old, 49 kg, 167 cm man (body mass index [BMI] of 17.6 kg/m^2^) was scheduled for transurethral resection of the prostate surgery due to the presence of prostatic hyperplasia. The patient had a 7-year history of T2DM and 5-year history of lumbar disc herniation. He had no prior hypoglycemia events. His medicines included saxagliptin 5 mg oral daily. He had good glycemic control with a fasting blood sugar of approximately 5 to 11 mmol/L, a post-meal blood sugar of about 6 to 15 mmol/L (Table 1), and a glycosylated hemoglobin of 6.9%. During hospitalization, his laboratory evaluations for hepatic function, renal function, and thyroid function were within normal ranges. A computed tomography scan of the abdomen revealed gallstones. No abnormalities were seen on the chest computed tomography or brain magnetic resonance imaging. The patient was instructed to stop saxagliptin the day before surgery and to fast after the dinner until the operation. At 8 am the morning of surgery, his fasting blood sugar was 4.7 mmol/L. Per the urologist’s order, the patient was given 500 mL of 5% dextrose solution and 5 units of insulin once, his blood glucose raised to 5.1 mmol/L at 10 am. The baseline blood glucose before starting anesthesia induction was 4.0 mmol/L at 12:53 pm. Preoperative blood pressure was 125/77 mm Hg and heart rate 73 bpm. The patient received intravenous etomidate (14 mg), sufentanil (15 μg), and cisatracurium (10 mg) for general anesthesia induction and was maintained with sevoflurane (1%–1.5%) and remifentanil (0.04–0.05 μg/kg/min). A laryngeal mask airway was placed for mechanical ventilation. Depth of anesthesia was monitored using bispectral index (BIS) monitoring. Twenty minutes after induction of general anesthesia, the BIS value was approximately 20 to 25. The patient’s hemodynamics were stable. Arterial blood sampling showed normal electrolytes and acid-base status but severe hypoglycemia with a blood glucose level of 0.98 mmol/L (Table 2). Arterial blood gas analysis at the time showed: pH 7.43, PaCO_2_ 39 mm Hg, PaO_2_ 214 mm Hg, HCO_3−_ 26 mmol/L, base excess 2 mmol/L, lactate 0.63 mmol/L. Repeat finger stick glucose testing gave a result of 0.96 mmol/L. Further venous blood samples were urgently obtained for a complete blood count, hepatic and renal function tests, coagulation tests, electrolytes, glucose, insulin, C-peptide and cortisol levels, then sent to the hospital laboratory; 40 milliliters of 50% dextrose were administered intravenously followed by an infusion of 5% dextrose. One hour later, the laboratory technologist reported the blood glucose was 0.45 mmol/L. The laboratory test results were as follows: low hemoglobin 8.4 g/L (normal 11–17.2 g/L), mild hypokalemia 3.4 mmol/L (normal 3.5–5.1 mmol/L), and hypoalbuminemia 26.5 g/L (normal 35–55 g/L). The remaining results were unremarkable including normal creatinine 82.1 mmol/L (57–111 mmol/L) and liver enzymes. Concurrent fasting insulin and C-peptide assays showed normal insulin secretion, with insulin at 2.1 μIU/mL (normal range: 2.0–28.0 μIU/mL) and C-peptide at 0.63 ng/mL (normal range: 0.28–2.86 ng/mL). His random cortisol level at 1:20 pm was 8.1 μg/dL (normal range: 6.7–22.6 μg/dL), excluding adrenal insufficiency (refer to Table 2). The patient’s hypoalbuminemia was likely due to inadequate oral intake during hospitalization. Ten minutes after treatment, the patient’s blood glucose was 5.3 mmol/L and the BIS was about 40. Twenty minutes after treatment, the glucose had risen to 10.7 mmol/L while the BIS ranged from 40 to 50. Recovery from anesthesia, extubation, and postoperative recovery were unremarkable. No further hypoglycemic episodes occurred during hospitalization. The patient had no neurological injury and was discharged home 4 days later. His neurological status remained normal at a 3-month follow-up. Written informed consent was obtained from the patient and his family for the publication of this case report and included data.

**Table 1:** Blood glucose levels during the hospitalization.

	Fasting blood glucose (mmol/L)	Post-meal blood glucose (mmol/L)
Day 1	5	8.7
Day 2	9.4	13–12.2–8.6
Day 3	5.4	5.9–10.6–5.7
Day 4	5.2	7.3–10.1–8.9
Day 5	5.5	8.2–7.7–9.5
Day 6	4.7–5.1–4.0–0.96	
Day 7	10.8	14.6–14.3
Day 8	7.2	12.2–12.4–12.1
Day 9	7.2	9.5–8.8

Day 1 indicates the day of of admission; Day 6 indicates the day of operation.

**Table 2:** Laboratory results of venous blood samples at hospital admission and during hypoglycemia occurrence.

	Hospital admission	Hypoglycemia occurrence	Normal ranges
Peripheral blood			
WBC (10–9/L)	6.63	12.95	3.7–9.6
RBC (10–12/L)	3.41	2.91	3.7–5.7
Hemoglobin (g/L)	9.8	8.4	110–172
Hct (%)	31.2	26.5	34–51
Blood chemistry			
Glucose (mmol/L)	5	0.45	3.89–6.11
Insulin (μIU/mL)		2.1	2.0–28.0
C-peptide (ng/ml)		0.63	0.28–2.86
TP (g/L)	74.9	52.6	60–85
Alb (g/L)	40	26.5	35–55
Potassium(mmol/L)	4.7	3.41	3.5–5.5
Sodium(mmol/L)	142	142	135–145
AST (U/L)	29	29	8–38
ALT (U/L)	16	12	0–45
Crea (mmol/L)	112	82.1	57–111
BUN (mmol/L)	8.19	4.41	2.7–8.2
Cortisol (μg/dL)		8.1	6.7–22.6
Arterial blood gas analysis			
pH		7.43	
PaCO_2_ (mm Hg)		39	
PaO_2_ (mm Hg)		214	
HCO_3−_ (mmol/L)		26	
Base excess (mmol/L)		2	
Lactate (mmol/L)		0.63	
Glucose (mmol/L)		0.98	

Alb = albumin, ALT = alanine aminotransferase, AST = aspartate aminotransferase, BUN = blood urea nitrogen, Crea = creatinine, RBC = red blood cells, TP = total protein, WBC = white blood cells.

## 3. Discussion

There are several potential causes of hypoglycemia in diabetic patients, including improper insulin dosing, concurrent medications, or deteriorating renal and liver function, adrenal insufficiency. The patient in this case was given a 500 mL infusion of 5% dextrose solution along with 5 units of insulin and missed 2 meals before surgery. The relative insulin overdose may have caused hypoglycemia during the operation. However, serum insulin and C-peptide levels checked during the hypoglycemia were within normal ranges. In this case, the hypoglycemia was unlikely due to insulin overdose. Adrenal insufficiency was ruled out based on normal cortisol hormone levels. The patient maintained stable renal and liver function throughout hospitalization, with no evidence of sepsis or starvation ketoacidosis. In the case we speculate that opioids were the culprits due to the temporal association and the rapid decline in blood glucose levels. Baseline blood glucose level before starting anesthesia induction was 4.0 mmol/L. However, it decreased substantially to 0.96 mmol/L immediately after anesthesia induction. And there were no hypoglycemic episodes during the 4-day observation period before discharge. Opioids have been reported to cause hypoglycemia through various mechanisms including increased glucose utilization by liver cells and skeletal muscles, promotion of insulin release, as well as impairment of the counter-regulatory response.^[7,8,11]^ Several hypotheses have been proposed for opioid-induced hypoglycemia, but remain controversial. A disproportionality analysis of the WHO global individual safety report database found opioid-induced hypoglycemia was likely a class effect.^[12]^ However, another retrospective analysis revealed a significant association of hypoglycemia with tramadol and methadone but not other opioids in nondiabetic patients.^[13]^ A recent review indicates that while opioid stimulation often elevates blood glucose levels, it can reduce levels in patients with type 2 diabetes.^[14]^ Diabetes appears to be a risk factor. Additionally, a case report described fasting hypoglycemia with acute, transient, opioid-induced secondary hypoadrenalism.^[15]^

We found no prior cases of opioids causing hypoglycemia immediately after anesthesia induction. The exact mechanism remains unknown. Animal studies show intrathecal morphine depletes hepatic glycogen in hypoglycemia.^[10]^ Other animal models demonstrate tramadol directly increases glucose utilization in hepatocytes and muscle through μ-opioid receptor activation in diabetic rats.^[16]^ We cautiously suggest opioids likely increased peripheral glucose uptake and impaired gluconeogenesis, causing acute hypoglycemia after induction. Compensatory mechanisms, such as glycogenolysis, are usually activated during hypoglycemia; however, this response may be diminished in patients with diabetes, prolonged fasting, low BMI, poor nutrition, or advanced age. All of these factors were present in our patient, possibly exacerbating the hypoglycemia. Such severe hypoglycemia in our case is explained by depleted hepatic glycogen reserves and gluconeogenesis substrates. We observed abnormally low BIS values during severe hypoglycemia. Two case reports describe intraoperative, abnormally low BIS values with severe hypoglycemia in diabetic patients.^[17]^ BIS may reflect severe hypoglycemia under general anesthesia to some extent.

### 3.1. Limitations

First, the imputation of hypoglycemia to opioids in our case is a conclusion inferred by our team after excluding other possibilities, based on relevant data and a review of previous literature. Second, as a case report, the generalizability of our findings is inherently limited. The particulars of this case and its distinctive circumstances limit the degree to which our results can be generalized to wider patient populations or situations. Therefore, caution should be used when applying these findings more broadly. Given these limitations, additional research with larger sample sizes and more comprehensive designs is necessary to confirm and validate the observed associations between hypoglycemia and opioids. By expanding the scope of inquiry, future studies may produce more robust and generalizable understandings of the relationships between opioids and hypoglycemic events.

## 4. Conclusions

With this report, we would like to highlight that patients exposed to opioids can experience severe hypoglycemia, especially those with diabetes undergoing general anesthesia, long fasting periods, low BMI, poor nutrition status, and advanced age. Blood glucose monitoring tends to be more frequent in the perioperative setting, particularly for diabetic patients under general anesthesia.

## Acknowledgments

We sincerely thank all the anesthesiologists and clinicians in our unit, who were involved in providing care and skilled management to the patient.

## Author contributions

Conceptualization: Qin Tian, Jia Lu Wan.

Data curation: Qin Tian, Ming Liang Yi.

Formal analysis: Qin Tian, Jia Lu Wan.

Investigation: Hong Yin, Ming Liang Yi.

Writing – original draft: Qin Tian.

Writing – review & editing: Hong Yin.
